# Long‐term evolutionary conflict, Sisyphean arms races, and power in Fisher's geometric model

**DOI:** 10.1002/ece3.5625

**Published:** 2019-09-04

**Authors:** Trey J. Scott, David C. Queller

**Affiliations:** ^1^ Department of Biology Washington University in St. Louis St. Louis MO USA

**Keywords:** adaptation, arms race, evolutionary conflict, Fisher's geometric model, joint phenotypes

## Abstract

Evolutionary conflict and arms races are important drivers of evolution in nature. During arms races, new abilities in one party select for counterabilities in the second party. This process can repeat and lead to successive fixations of novel mutations, without a long‐term increase in fitness. Models of co‐evolution rarely address successive fixations, and one of the main models that use successive fixations—Fisher's geometric model—does not address co‐evolution. We address this gap by expanding Fisher's geometric model to the evolution of joint phenotypes that are affected by two parties, such as probability of infection of a host by a pathogen. The model confirms important intuitions and offers some new insights. Conflict can lead to long‐term Sisyphean arms races, where parties continue to climb toward their fitness peaks, but are dragged back down by their opponents. This results in far more adaptive evolution compared to the standard geometric model. It also results in fixation of mutations of larger effect, with the important implication that the common modeling assumption of small mutations will apply less often under conflict. Even in comparison with random abiotic change of the same magnitude, evolution under conflict results in greater distances from the optimum, lower fitness, and more fixations, but surprisingly, not larger fixed mutations. We also show how asymmetries in selection strength, mutation size, and mutation input allow one party to win over another. However, winning abilities come with diminishing returns, helping to keep weaker parties in the game.

## Graphical abstract

We extend Fisher's geometric model to instances involving evolutionary conflict, where two parties have divergent evolutionary interests. We compare this extension to more traditional models and investigate characteristics that allow one side to win the arms race.

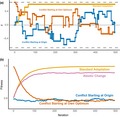

## 1 INTRODUCTION

Through natural selection, species may become adapted to their environments. Environments include interactions with other organisms, both within and between species. When there is evolutionary conflict, that is when each party can increase its fitness at the expense of the other party, this process of adaptation can drive antagonistic co‐evolution and arms races, leading to maladaptation of one or both parties (Brockhurst et al., 2014; Dawkins & Krebs, 1979; Queller & Strassmann, 2018; Van Valen, 1973). These interactions may be important drivers of evolution in nature (Queller & Strassmann, 2018; Thompson, 2013). Evidence that this is so includes rapid adaptation in response to new biotic foes (Reznick & Ghalambor, 2001), molecular evolution in response to pathogens (Enard, Cai, Gwennap, & Petrov, 2016; Endo, Ikeo, & Gojobori, 1996; Tiffin, 2004), and the many examples of arms races found in nature (Benkman, Parchman, Favis, & Siepielski, 2003; Berenbaum & Zangerl, 1998; Decaestecker et al., 2007; Edger et al., 2015; Hanifin, Brodie, & Brodie, 2008; Toju, 2008) and evolved in the laboratory (Pal, Maciá, Oliver, Schachar, & Buckling, 2007; Paterson et al., 2010). This evidence supports the idea (Dawkins & Krebs, 1979; Van Valen, 1973) that conflicts may drive a great deal of adaptive evolution, though without necessarily leading to higher fitness.

Conflict has been analyzed in various models of co‐evolution (Kokko, Jennions, & Brooks, 2006; Nuismer, 2017). However, Fisher's (1930) geometric model is conspicuously absent from studies of co‐evolution. This is surprising because of the geometric model's success as a general model of adaptation and because of its focus on successive fixations (Tenaillon, 2014), an essential element of many arms races (Daugherty & Malik, 2012; Edger et al., 2015; Marston et al., 2012; Woolhouse, Webster, Domingo, Charlesworth, & Levin, 2002) that is usually left out of co‐evolutionary models (unless changes are assumed to be very small; e.g., Dieckmann & Law, 1996; Nuismer, 2017). The ability to model successive fixations should make the geometric model a potentially powerful tool to investigate how conflict can lead to arms races.

**Figure 1:**
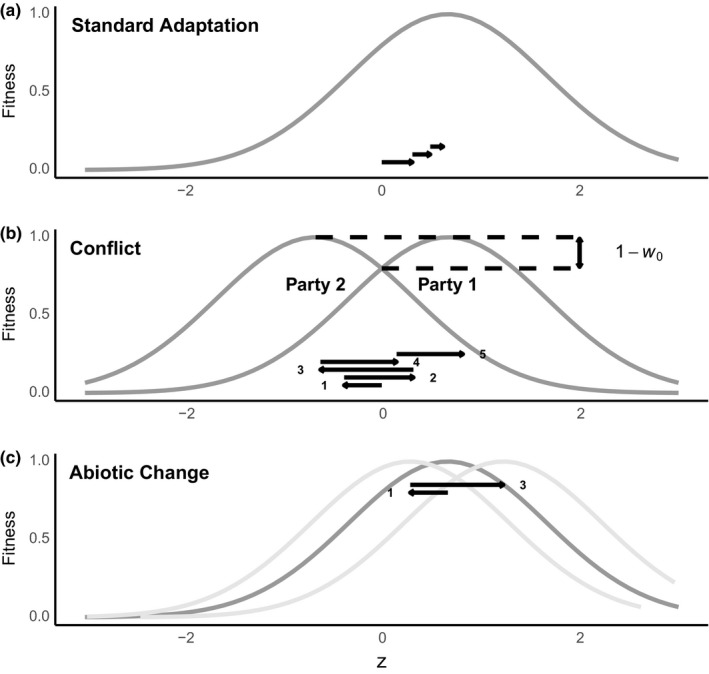
Schematic diagrams of the three versions of Fisher's geometric model studied: standard adaptation, conflict, and a moving optimum. Each panel shows one or more fitness curves as a function of a trait value z and some fixations (arrows). (a) In a standard geometric model, parties adapt a trait *z* to a single stable optimum by fixing beneficial mutations. (b) In models with conflict, *z* represents values of a joint phenotype, and there are two fitness functions, corresponding to party 1 and party 2 (we arbitrarily assign party 1 the positive optimum value). Conflict is measured by the lag load, 1−*w*_0_, at the point of intersection for both fitness functions. Parties can fix beneficial mutations back and forth (arrows), with party 1 fixing 2, 4, and 5 and party 2 fixing 1 and 3 in this example. (c) Abiotic environmental change is modeled by shifting a single party's optimum in a random direction. In order to compare changes of equal size to the biotic conflict scenario, in each iteration we shift the optimal value of trait *z*, in a random direction, by the same amount that party 1 experiences biotic environmental change in the conflict simulation—that is, by the amount that antagonistic party 2 changes *z* (fixations 1 and 3)

Fisher's geometric model treats an adapting population as an *n*‐dimensional vector of trait values. Somewhere in *n*‐dimensional space is an optimum where the population is most fit for all *n* traits. In an initially monomorphic population, a random mutation is introduced, typically from a Gaussian distribution, causing an additive shift in the trait space. This mutation can either fix or be lost, making the population monomorphic again. Selection favors mutations that move the population closer to the optimum (Figure 1a shows a single‐trait version of this process). By this process of successively fixing mutations, a population goes on an “adaptive walk,” usually from lower fitness to higher fitness closer to the optimum (Tenaillon, 2014). Large populations in Fisher's geometric model move relatively rapidly to the optimum and stay there. With small population sizes, fixations of small deleterious mutations due to drift keep a population at a variable, but usually small, distance from the optimum (Poon & Otto, 2000). An important result from studies of these adaptive walks is that mutations are less likely to fix when a population is well adapted, and this is especially true for mutations of large effect (we will call these large mutations) because they can overshoot the optimum (Fisher, 1930; Orr, 1998, 2005).

To capture evolution under biotic conflict with Fisher's geometric model, we need to consider two evolving parties instead of one. In order to link them, we use the concept of a joint phenotype, a trait that is the shared outcome of the actions of two different parties (Queller, 2014; Queller & Strassmann, 2018). For example, we could think of the probability of infection as a joint phenotype; various traits of the pathogen and host may interact to make the joint phenotype, and evolution in either party can change the value of this phenotype. Other examples of joint phenotypes are the probability that a predator catches a prey in a given encounter and the amount of food a cuckoo chick gets from its host. More formally, joint phenotypes are a general way to model interactions between different parties, where the interaction between two traits *x* and *y* is reduced to a single measure *z* = *f*(*x*, *y*). Thus, joint phenotypes include more commonly used interaction models, such as those that are determined by the difference between traits *z* = *x* − *y* (Nuismer, 2017).

Joint phenotypes are often high‐level phenotypes in the sense that they can be affected by many lower‐level private phenotypes or traits, that is, traits that belong to one of the two parties. The joint phenotype emerges from the net effect of interactions between lower‐level private traits. For the predator and prey, these private traits might include speed, agility, stealth, sensory capabilities, weapons, and defenses (with still lower‐level phenotypes contributing to each of those). The joint phenotype is the net effect—the probability a prey is caught by a predator. This kind of multidimensionality must be important in many arms races but is only beginning to be studied (Débarre, Nuismer, & Doebeli, 2014; Gilman, Nuismer, & Jhwueng, 2012).

Two parties experience potential conflict (Ratnieks & Reeve, 1992) when selection is expected to pull the joint phenotype in opposite directions, that is, whenever the trait value lies between the optimal trait values for the two parties (Queller, 2014; Queller & Strassmann, 2018). In this region, a change in the trait in one direction usually benefits one party but harms the other. The evolution of joint phenotypes has been explored in a quantitative genetic context, including derivation of a conflict version of Fisher's fundamental theorem of natural selection (Queller, 2014). Adding joint phenotypes into Fisher's geometric model offers a straightforward way to model conflict evolution via fixation of successive novel mutations. Here, we examine the simplest case, a one‐dimensional (single trait) model of conflict in which the two parties have different optima. This case corresponds to the evolution of a joint trait that is uncorrelated with other traits.

Based on what is known about adaptive walks in the standard geometric model, we can make some predictions about how the model might behave with conflict over a joint phenotype (Queller & Strassmann, 2018). Both parties should fix mutations that move them closer to their respective optima. However, a novel feature is that when one party fixes a mutation, it should usually pull the second party away from its optimum. Returning to the example of host–pathogen conflict, a pathogen may evolve a new receptor that increases its ability to infect the host. The joint phenotype—probability of infection—is now closer to the pathogen's optimum and farther from the host's. There should now be strong selection for the host to evolve a counter for the new receptor and move the probability of infection closer to its optimum. This adaptive process can repeat as long as there are new mutations and neither party has gone extinct or ceased to interact in a way that can be modeled as a joint phenotype.

On average, the joint phenotype should therefore lie somewhere between the optimal values of the two parties. In agreement with the Red Queen hypothesis (Van Valen, 1973), each party should run more or less in place just to maintain its fitness. However, they should not stay exactly in place; each party in an arms race should push the trait in a direction that increases its own fitness, but always be dragged back down by the other party. We call these Sisyphean dynamics, after Sisyphus who, in Greek mythology, was condemned to forever push a boulder up a hill, only to have Zeus roll it back down.

Because parties with conflict should be farther from their optima, we expect two standard outcomes of Fisher's original model under that condition: higher fixation probability and larger fixed mutations. More mutations should fix because being far from the optimum enlarges the space of beneficial mutations. When the population is close to the optimum, some large mutations in the right direction should be disfavored because they overshoot the optimum, but this should happen less when the population is far from the optimum.

We also expect that conflict may be more pernicious than a changing abiotic environment (Connallon & Clark, 2015; Kopp & Hermisson, 2009a, 2009b). Both kinds of changes can cause a mismatch between trait value and optimum value, but once sufficiently far away from the optimum, the two kinds of changes may have different effects. A random abiotic change may often alter the optimum in a beneficial direction, up to about half the time when the change is small, but a change due to a conflicting party should usually be nonrandom in the direction away from the optimum.

Extending previous modeling (Gandon & Michalakis, 2002), we also explore how parties can gain an advantage during co‐evolution because of various asymmetries, such as population size, relative input and effect size of mutations, and the strength of selection (the dinner–life principle (Dawkins & Krebs, 1979)).

## 2 METHODS

In order to illustrate the most fundamental properties of the geometric model with conflict, we study the simplest possible version of the model, with a single trait and with two haploid parties that differ in their optima for that trait. We begin with a single axis corresponding to a phenotypic trait, shared or influenced by two parties, whose value is represented as *z*. The two parties could be either different species or different roles within species, such as males and females.

Fitness is assumed to be a Gaussian function of a party's distance from its optimum, *o_i_*, where *i* indexes parties to the conflict and takes the values 1 or 2 for a two‐party conflict:

$$w_{i}=e^{-\omega_{i}{\left(o_{i}-z\right)}^{2}}.$$

At *o_i_*, fitness is equal to 1 for party *i*, but falls off in both directions. The shape parameter *ω_i_* determines how quickly fitness falls off for each party. This can be viewed as a phenomenological function that will not necessarily exactly match the ecologies of particular interacting species. But it serves to broadly represent the general idea of fitness optima, and it is necessary to provide comparable results that can be compared with the standard nonconflict Fisherian model.

Figure 1b shows fitness functions for two parties, in this case having the same *ω*. The point of intersection is interesting because it is the value of *z* at which the two parties have equal fitness, *w*_0_, and therefore equal reductions in fitness or lag loads, defined as 1 − *w*_0_ (Maynard Smith, 1976). We will use this shared value of lag load as a convenient summary measure of degree of conflict (though other measures of conflict are possible). It describes how much each party stands to gain by moving from this point to its optimum (Figure 1b). By varying the lag load in the model, we adjust the intensity of conflict and the distance between the optima. For convenience, we define the point where the fitness functions intersect as the origin. Solving the fitness function for *z* = 0 gives two optima, $o_{i}=\pm \sqrt{-\ln\left(w_{0}\right)/\omega_{i}}$ showing that, for a given shape parameter value, increasing the shared lag load 1−*w*_0_ increases the distance between the optima.

We assume selection is strong relative to mutation, such that selection acts on a single mutation at a time. The model consists of steps that consist of drawing a mutation in one party and then determining whether the mutation will be fixed by selection. If so, it becomes a fixation and changes the value of the trait, affecting the fitness of both parties. The process is then repeated (in our initial models by alternating mutations between parties). The model thus assumes a separation of timescales of mutations and selective sweeps between parties; only one fixation occurs at a time.

The effects of mutations in the phenotypic space are assumed to be unbiased and normally distributed, $m_{i}∼N\left(0,\;\sigma_{i}\right)$, where *σ_i_* is the standard deviation (but see Figure 4c and Supplement for other distributions). Since mutation sizes are always positive, *σ_i_* determines the average size of mutations ${\bar{m}}_{i}=\sigma_{i}\sqrt{2}/\sqrt{\pi }$. We will vary this parameter to compare scenarios where mutations are very small relative to the distance to the optimum with cases where a party could reach its optimum with a single fixation.

In order to focus on the most basic features of conflict, we first examine the simplest possible case where the two partners are identical in every respect except their fitness optima *o*_1_ and *o*_2_ before moving on to models with asymmetries. Thus, we initially let the shapes of their fitness curves be the same (*ω*_1_ = *ω*_2_ = 1/2) and also let the mutation distributions be the same (*σ*_1_ = *σ*_2_). We drop subscripts for *σ* and *ω* when both parties have the same values. The number of new mutations will also initially be assumed to be the same for each party; in that, each iteration of a simulation consists of a round of mutation and possible fixation (adaptation) for each party. A randomly chosen party mutates first each iteration, followed by the second party.

We later relax these assumptions to test whether asymmetries in evolutionary potential, for example in selection strength or mutational input, allow one party to win over the other. To change the strength of selection, we define a relative selection strength parameter *f* that increases selection on party 1 relative to party 2 (*ω*_1_ = *fω*_2_). We manipulate the relative mutation sizes by giving party 1 *κ*‐fold larger average mutation sizes than party 2 (${\bar{m}}_{1}$ = *κ* ${\bar{m}}_{2}$). Lastly, we define a relative mutational input parameter *r*, where party 1 will generate (and potentially fix) *r* mutations for every 1 mutation of party 2 (*µ*_1_ = *rµ*_2_). This mutational input parameter would include effects of population size, mutation rate per generation, and number of generations.

To measure “winning” during conflict, we use the idea of fitness power (Queller & Strassmann, 2018). Fitness power for party 1 is defined as *P_w_*_1_ = 1 − *L*_1_/(*L*_1_ + *L*_2_), where *L_i_* is the lag load (1 − *w*_0_) for party *i*. This power value ranges from 0 to 1, where values closer to 1 mean that party 1 is winning the conflict in terms of being closer to its fitness maximum, a value of 0.5 means that both parties have equal lag loads, and power <0.5 means that party 2 is winning.

Unless stated otherwise, we assume that both parties are in very large populations where the effect of drift is tiny, so the probability of fixation is $\Pi_{i}=1-e^{-2s_{i}}$ for positive values of *s_i_* ($\Pi_{i}=0$ for *s_i_* < 0), where *s_i_* is the selection coefficient for a new mutation (Kimura, 1983). For smaller population sizes, *N_i_*, the probability of fixation is $\Pi_{i}=\left(1-e^{-2s_{i}}\right)/\left(1-e^{-4N_{i}s_{i}}\right)$ for both positive and negative values of *s_i_* (Kimura, 1983). The selection coefficient is $s_{i}=w_{m}/w_{i}-1$, where *w_m_* is the fitness of a new mutation and *w_i_* is the population's current fitness.

For comparison, we examine a single population with one trait adapting to its optimum without conflict—the standard geometric model (Figure 1a). Our nonconflict control populations are always assigned the same parameter values as party 1 in the corresponding conflict scenario.

Each simulation consists of many iterations of the mutation and selection process to model an adaptive walk. When our interest is in equilibrium conditions, we first eliminate data from 250/*ω* iterations, in which simulations showed to contain the initial period of rapid adaptation for the standard model. After this initial adaptation period, we calculate average results over the next 5,000 iterations for 1,000 replicate simulations (for a total of 5,000,000 iterations for each condition simulated). We expect that conflict will result in populations that are often away from their optima, resulting in more fixations of larger phenotypic effect (which we will simply call larger fixations).

We also expect conflict to be more detrimental to fitness than simulations involving a randomly changing abiotic environment, primarily because more changes due to a conflicting party should be away from the optimum. To test this, we need a nonconflict control simulation with nondirectional changes of the same magnitude as changes in the conflict simulation. This is complicated by the fact that the changes are of different types in the two scenarios. In our conflict model, what changes is the joint phenotype, while the optimum remains the same (the pathogen's joint phenotype changes when hosts evolve more resistance, but its optimum is still to have a high probability of infection). In models of abiotic change (Connallon & Clark, 2015; Gordo & Campos, 2012; Matuszewski, Hermisson, & Kopp, 2014), the reverse is typically assumed; the environmental change does not alter the phenotype but does alter the optimum. But common currency can be found because both shift the distance between trait and optimum along the *z*‐axis. Therefore, for each conflict simulation, we simulated a parallel single‐population moving‐optimum model, where the environment changes the optimum in every iteration by exactly the same amount (including no change) that party 1's opponent changes the joint phenotype in that same iteration of the conflict simulation, but in a random direction (Figure 1c). Thus, both conflict and abiotic change simulations involve the same magnitude of change, but we expect that the effect on the joint phenotype may usually be deleterious for party 1 while the effect on the abiotic optimum may be deleterious or beneficial.

## 3 RESULTS

**Figure 2:**
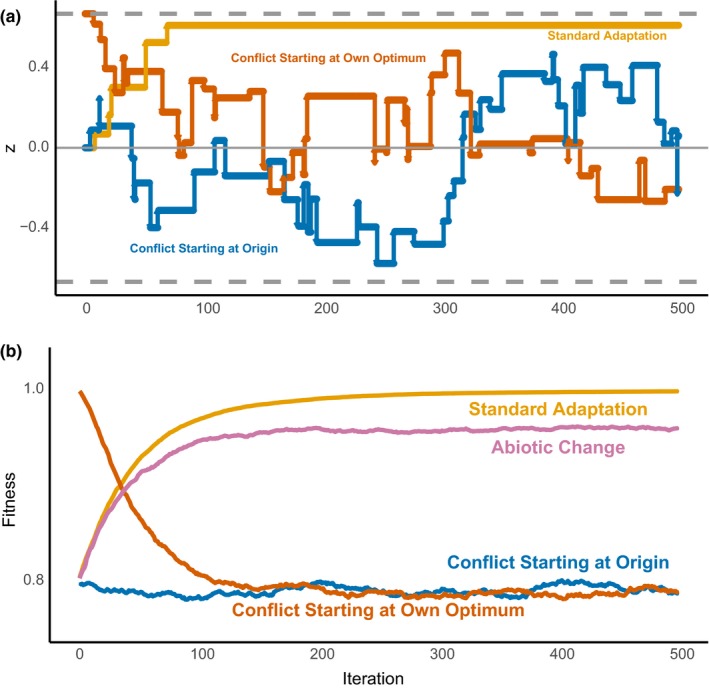
The effect of conflict on adaptive walks and fitness trajectories. (a) Three adaptive walks of mutations fixed with and without conflict. We include a standard model that adapts to a single optimum and models with conflict that begin at either the origin or at party 1's optimum. Horizontal dashed lines indicate the optimal phenotypes of the two parties. (b) Fitness trajectories based on averaged fitness from 1,000 simulated adaptive walks of each type. For all simulations, both parties had conflict intensity values of 0.2, average mutation sizes of 0.1, a fitness function shape parameter of 1/2, and infinite populations

A sample simulation shows, in accord with prior results (Tenaillon, 2014), that in the standard geometric model, a single party fixed many mutations initially and then stabilized near the optimum (Figure 2a). But with conflict, regardless of whether the simulation started at the origin or elsewhere, there was no straightforward walk to a stable point. Instead, populations contested the value of the joint phenotype, resulting in back‐and‐forth Sisyphean movement of the joint phenotypic value (Figure 2a). Conflict parties constantly fixed adaptive mutations but the improvement was canceled out by changes due to the other party, as proposed by the Red Queen hypothesis (Van Valen, 1973).

We investigated how these dynamics affected fitness when averaged over 1,000 adaptive walks (Figure 2b). In the standard model, parties increased fitness over time and then stayed near the maximum fitness of 1 (in finite populations, chance fixation of deleterious mutations could allow small movements away from the optimum (Poon & Otto, 2000)). In contrast, parties with conflict were far from the maximum fitness. We also show the fitness trajectory for a party adapting to random abiotic change that is equal to the magnitude of change due to fixations from an antagonistic partner. This example showed more rapid adaptation and maintenance of higher fitness under abiotic change than under conflict.

To better understand how conflict affects long‐term evolution, we compared the equilibrium properties, after the initial period of rapid adaptation, of replicate conflict and nonconflict simulations for different combinations of parameters.

**Figure 3:**
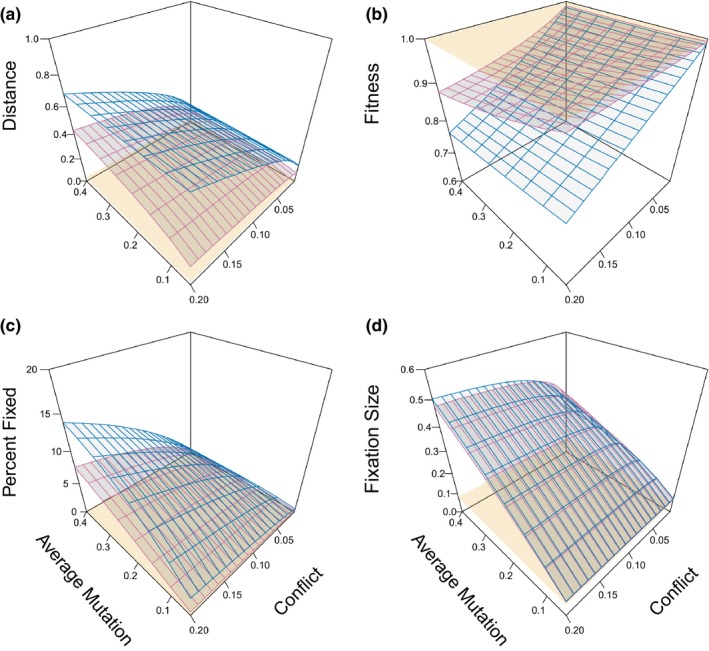
Equilibrium properties of the geometric model under varying average mutation sizes (normally distributed) and conflict intensities (measured as lag load at the origin where the fitness functions intersect (1−*w*_0_) from fitness functions with a shape parameter of 1/2). Colors indicate the version of the geometric model: standard adaptation (yellow), conflict (blue), and abiotic change (pink). (a) Mean distance to the optimum. (b) Mean fitness during equilibrium. (c) Percent of mutations that are fixed during equilibrium. (d) Effect size of fixed mutations. Means of the independent variables are calculated based on data collected from iteration 500–5,500 from 1,000 replicate simulations with infinite populations. Vertices show actual mean values from simulations

In contrast to parties under the standard model, which essentially went to their optimum values (Figure 3a, yellow), parties with conflict (blue) and abiotic change (pink) were generally away from their optima though, as predicted, parties adapting to abiotic change approached their optima more than conflicting parties. Fitness is essentially maximized for standard adaptation parties without conflict after the period of rapid adaptation, but, as expected, parties with conflict have decreased fitness relative to standard adaptation (Figure 3b). Parties with conflict were largely stuck near their intersection fitness values defined by the lag load. Abiotic change also resulted in reduced average fitness but, as we predicted, to a lesser intensity than conflict. This difference was greatest when movement away from the optimum is strongest (high conflict) and movement toward it is weakest (low mutation size).

In standard adaptation simulations, few mutations were fixed (after the period of initial adaptation) because the party was close to, or at, the optimum value. Conflicting parties fixed more mutations, especially with larger mutation sizes (Figure 3c). Similarly, increased conflict resulted in more fixations. Abiotic change simulations showed a similar pattern to conflict simulations, but with fewer overall fixations.

The average phenotypic effect of fixation (fixation size for short) was larger under conflict and abiotic change compared to fixations from standard adaptation (Figure 3d), an effect previously shown for abiotic change (Kopp & Hermisson, 2009a, 2009b). This is expected because being away from the optimum decreases the likelihood that large mutations will overshoot the optimum. However, under this logic one would expect that the conflict case would fix larger mutations than the abiotic one and, interestingly, this is not the case (Figure 3d).

Up to this point, we have assumed that mutations are normally distributed and that the shape parameter, *ω*, is 1/2. We investigated whether our results were robust to changes in these assumptions (see online supplement; Figures S1–S4). Setting the mutation distribution to be either uniform or exponential did not notably impact our results (Figures S1 and S2). Similarly, normal fitness functions that are fourfold narrower (*ω* = 2) or wider (*ω* = 1/8), respectively, gave qualitatively similar results to those when *ω* is 1/2 (Figures S3 and S4). This is unsurprising since changing the strength of selection is equivalent to changing the sizes of mutations.

**Figure 4:**
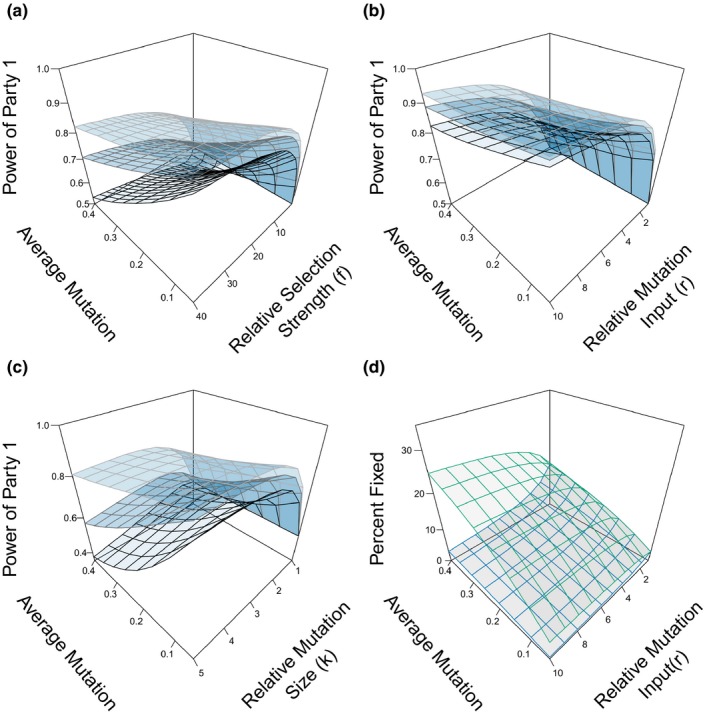
Fitness power is usually greater for the party with higher selection strength, mutational input, or mutation size. Fitness power was calculated according to *P_w_*_1_ = 1 − *L*_1_/(*L*_1_ + *L*_2_), where *L_i_* is the average lag load for party *i*. Intensity of conflict was 0.2, and population sizes were infinite for all simulations. (a) Fitness power for party 1 when *ω*_1_ = 2 (black), 1/2 (gray), or 1/8 (light gray) and *ω*_2_ = *ω*_1_/*f*, where *f* is the relative selection strength. Each party has one mutation per iteration. (b) Fitness power for party 1, where party 1 generates r times more mutations than party 2. Colors correspond to the same *ω*_1_ values as shown in A, but *ω* is the same for both populations. (c) Fitness power for party 1 when ${\bar{m}}_{1}$ = *κ* ${\bar{m}}_{2}$, where *κ* is the relative mutation size. Each party has one mutation per iteration. Results are shown for different values of *ω*. The average mutation axis shows the average mutation size for party 2 (${\bar{m}}_{2}$). (d) Percent of mutations that are fixed for party 1 (blue) and party 2 (green) with varying relative mutation inputs and mutation sizes. Average power is calculated from 5,000 iterations as outlined in the text. Vertices show actual mean values from simulations

We also investigated whether asymmetries in factors affecting adaptive potential allow one party to win the conflict using the measure of fitness power defined earlier (see Methods). One factor that considerably affected power was the relative selection strength, *f*. When party 1 is under stronger selection (*f* > 1), it also had greater power though with diminishing returns for larger values of *f* (Figure 4a). There was an interaction with mutation size, where power tended to decrease with larger average mutation size, especially when selection strength (*ω*) was high (Figure 4a). Large mutations in the party close to its optimum often overshoot, whereas for the losing party they offer a chance to get close to its optimum quickly.

Power was greater when a party had a higher mutational input, which can be due to this greater population size, mutation rate per generation, or number of generations. When party 1 generated more mutations for every mutation of party 2, the fitness power for party 1 was higher (Figure 4b). This effect leveled off with increasing mutation rate and was largely independent of mutation size and *ω*. In our model, the mutational input was controlled by a single parameter, but in real populations, it could differ through a difference either in mutation rates or in population sizes so our result indicates that either of these can increase power.

A distinct effect of population size, the effect of drift on fixation, was tested separately in our model. Given the same total mutational input, larger populations should fix more beneficial mutations and fewer deleterious ones. However, this resulted in only a slight increase in power even when one population is very small (*N* = 10; Figure S5).

Higher relative selection strength and higher relative mutational input always gave party 1 higher power (>0.5) under the parameter values we explored, and so did higher relative mutation size in most, but not all, parameter combinations. Increasing the relative mutation size for party 1 when party 2 generated small mutations resulted in higher power for party 1 (Figure 4c). However, increasing party 1's relative mutation size when party 2 generated large mutations resulted in lower power because of the increased tendency to overshoot the optimum. At the most extreme values, this reduction in power actually results in party 2 winning the conflict as shown by power values below 0.5.

Winning and losing parties tended to fix different amounts and sizes of mutations as a result of being closer or farther from the optimum (Figure 4d). Winning parties tend to fix fewer and smaller mutations, moving close to the nonconflict case (Figures S6–S8), while losing parties tend to fix more and larger mutations (Figures S9–S11).

Finally, we note that asymmetries did not alter our qualitative conclusions (Figure 3) about differences from abiotic change or the standard model. When party 1 is given higher or lower selection strength (Figures S6 and S9), mutation size (Figures S7 and S10), or mutational input (Figures S8 and S10), it still has larger distances to the optimum, lower fitness, more fixations, and larger fixations than parties with the same parameters under abiotic change and standard adaptation.

## 4 DISCUSSION

One cannot fully understand adaptation without also understanding maladaptation. Previous modifications of the geometric model allowed it to model maladaptation due to factors such as genetic drift and environmental changes to optima (Kopp & Hermisson, 2009a; Poon & Otto, 2000). But it remained largely silent about what may be the major source of maladaptation, evolutionary conflict between organisms (Queller & Strassmann, 2018). By introducing joint phenotypes into Fisher's geometric model, we have expanded a powerful model of long‐term adaptation by successive fixations to the study of conflict and arms races, where such successive fixations are likely to be especially important. This approach erases a major shortcoming of the geometric model and brings the power of the geometric model to bear on co‐evolution.

Our results confirm the longstanding view that conflict and a succession of de novo mutations can engender long‐term arms races (Dawkins & Krebs, 1979). Whereas the standard model rapidly approaches its optimum and largely stops evolving, both parties under conflict are held off their optima (Figure 3a), suffer decreased fitness (Figure 3b), and consequently continue to fix numerous mutations (Figure 3c). This running rapidly to stay in the same place is what is expected under the Red Queen hypothesis (Van Valen, 1973) with the qualification that the “same place” is a long‐term average of Sisyphean advances and rollbacks.

Another interesting result from this work is that conflict has more severe effects on average fitness than does a randomly changing abiotic environment, when the changes are forced to be of the same magnitude (Figure 3b). This is largely because abiotic changes are modeled as changing the optimum in a random direction, whereas environmental change in the form of evolution of an opposing party naturally tends to change the joint trait in a malevolent direction. Our model allows abiotic change to extend indefinitely. However, in nature, abiotic changes may often vary around, and tend to return to, some central value. This would tend to reduce abiotic maladaptation, making the difference from biotic factors even starker than in our simulations. This supports the intuitive idea that conflict is a distinctly detrimental type of interaction that plays a powerful selective role, although the model cannot address whether abiotic or biotic changes in nature are larger in magnitude.

Some of these results are intuitive extensions of the standard model with the modification that the population is kept off its optimum (Queller & Strassmann, 2018). However, most of the results also show less‐than‐obvious nonlinear interactions among the variables. The most surprising concerns the size of fixations (Figure 3d). Being farther from the optimum should allow fixation of larger mutations to be fixed, and we see that is true for both the conflict and abiotic models relative to the standard model. But parties in conflict and parties adapting to abiotic change fix roughly the same sizes of mutations, despite parties with conflict being farther from the optimum. The reason appears to lie in the relationship between mutation size, conflict, and distance to the optimum (Figure 3a). When conflict is low, distance to the optimum is always low in both biotic and abiotic simulations, so there is no major difference in fixation size. If conflict is high (back right face of Figure 3a), distances to the optimum are larger, but in an interesting way. When mutation sizes are small, then a conflict party stays at substantially greater distances from the optimum than a party adapting to abiotic change. That would appear to open the door for larger fixations for the conflict case, but it does not because mutation sizes are too small—there are very few mutations large enough to fix in the conflict case but not in the abiotic. On the other hand, if mutation sizes are large relative to conflict, then the distances from the optimum are not so different for the conflict and abiotic cases and so fixation sizes are also not too different.

We also found both familiar and novel results when investigating how asymmetries during conflict can allow one party to win. First, by varying the fitness functions between parties, we were able to investigate whether stronger selection can favor one party over another. This could include the life–dinner principle, in which one party pays more dearly for losing (Dawkins & Krebs, 1979), and the rare‐enemy effect, in which individuals of one party experience the other less often (Dawkins, 1982). We find that a party with stronger selection does have higher fitness power, but that this effect is diminished with larger mutation sizes (Figure 4a). Stronger selection means a more narrow fitness function, which increases the probability that large mutations will overshoot. Thus, when mutations are large, stronger selection can be a disadvantage. However, our model does not include the possibility that strong selection could drive a population extinct.

Differences in mutational input—which could arise through differences in mutation rate, population size, or generation times—are an important parameter for winning an arms race (Figure 4b). Mutation rates have been shown to be important for winning in matching allele models (Gandon & Michalakis, 2002) and in experiments with bacteria (Pal et al., 2007). Our results broaden that conclusion to the case where all evolutionary change is due to de novo mutations, which is more likely in bacteria.

Larger populations have also been shown to be advantageous (Gandon & Michalakis, 2002). Our results show that this is true to the extent they increase mutational input, but the other potential advantage—of weaker drift—is generally very small (Figure S5).

Mutations play another role in determining the winner of an arms race through the sizes of mutations available to a party. We found that larger relative mutations increase power as long as mutations are not so large that they consistently overshoot the optimum (Figure 4c). This result suggests that larger mutational neighborhoods and increased “evolvability” may be advantageous during conflict, provided they do not increase the chances of overshooting the optimum.

Interestingly, none of our results show one party winning absolutely. Instead, increases in power tend to saturate with increases in selection, mutational input, drift, and mutation size (Figure 4a–c). This appears to be a result of the adaptive process described by the geometric model. The more power a party has, the more it will approach its optimum and decrease its pool of beneficial mutations. Because the second party is farther from its optimum, its pool of mutations will increase, leading to larger and more frequent fixations (Figure 4d, Figures S6–S11). This means that adaptation saturates for the winning party.

A similar dynamic works when two factors increase power; if party 1 has a greater evolutionary potential from one factor, it usually gets less added benefit from another factor. For example, when party 1 has greater potential in terms of selection strength (Figure 4a, right axis) and then mutation size is increased equally for both parties (Figure 4a, left axis), party 1's advantage is diminished (it still wins, but not by as much). Because party 1 is closer to its optimum owing to greater selection strength, it has less room to improve and is less able than its partner to take advantage of the equal increase in mutation sizes.

This saturation of power for the stronger party reflects a force that tends to keep weaker parties in the game. But this is not absolute. Fisher's model does not include population dynamics and the possibility that a strong partner will drive its antagonist to extinction. More explicit eco‐evolutionary models would be needed to address this question.

Arms races and Red Queen evolution have been classified into three types: fluctuating, escalatory, and chase (Brockhurst et al., 2014). The first three columns of Table 1 list some of their characteristics, modified from Brockhurst et al. (2014), and the fourth column lists the characteristics of the kind of arms race we have modeled. We call the new arms race Sisyphean, to emphasize the constant pushing of the trait uphill only to have it roll back.

**Table 1:** Types of arms races

Trait dynamics	Fluctuating	Escalatory	Chase	Sisyphean
Possible example	Daphnia and their pathogens^a^	Garter snakes and toxic newts^b^	Crossbills and lodgepole pine^c^	Cuckoos and their hosts^d^
Genetic architecture of traits	Few major loci	Polygenic or quantitative trait	Polygenic or quantitative trait	Successive single‐gene fixations
Basis of trait interaction	Matching to partner's trait	Excess over partner's trait	Matching to partner's trait	Tug‐of‐war over joint trait*
Selection mode	Fluctuating	Directional (unidimensional)	Directional (multidimensional)	Directional (multidimensional)
Allele frequency dynamics	Oscillations	Selective sweeps	Selective sweeps	Selective sweeps
Adaptive landscape	Multiple fitness optima	Fixed fitness optimum	Shifting fitness optimum	One fixed fitness optimum for each party

Note

Adapted and expanded from Brockhurst et al., 2014.

a Decaestecker et al. (2007).

b Hanifin et al. (2008).

c Benkman et al. (2003).

d For more examples, see Queller and Strassmann (2018).

* Same as joint phenotype in this paper; “trait” is used here for consistency with other entries.

The key differences in Sisyphean arms races are in the kind of trait evolving and in the timescale on which evolution is followed. Sisyphean arms races are best understood through joint phenotypes where two parties have different optimal values, as opposed to the separate private traits each with a single optimum in more traditional co‐evolutionary models. This joint phenotype is a general way to conceptualize conflict that does not require specification of the private traits (Queller & Strassmann, 2018). The trait interaction is based on a tug‐of‐war over the joint phenotype. The tug‐of‐war metaphor has often been used for more specific cases, for example, over the joint phenotype of offspring provisioning (Haig, 1993; Moore & Haig, 1991) or use of group resources for reproduction (Reeve, Emlen, & Keller, 1998; Shen & Kern Reeve, 2010). The joint phenotype is the object of the tug‐of‐war, and in Sisyphean arms races, the tug‐of‐war occurs over long timescales via successive fixations. We can thus differentiate arms races on the short end of the continuum, like fluctuating arms races with recurring changes in frequencies of the same set of genes, from Sisyphean arms races, where change happens on longer timescales and is mediated by successive fixations of different genes resulting in fluctuating joint phenotype values.

The boundaries among the types of arms race in Table 1 are not always clear‐cut. In fact, sometimes other arms races, which consider only private traits, could rescale into Sisyphean arms races when we consider the joint phenotype over long periods of time. For example, an escalatory arms race between seed hardness and beak strength of a bird is also a Sisyphean arms race over the probability that the seed gets eaten. Likewise, the individual color and pattern traits of a butterfly mimic may evolutionarily chase those of its model, but this is also a Sisyphean arms race over the abstract joint trait of degree of similarity, with the mimic having an optimum at high similarity and the model having an optimum at very low similarity (although our particular model may need to be adjusted because it assumes fitness falloffs on both sides of the optimum).

Biologically, complex Sisyphean arms races are more likely to entail long‐term persistent antagonistic evolution. Strong selection on a single trait, like cheetah speed, might ultimately deplete it of possible beneficial mutations, but this is less likely for a joint trait with many subtraits. Moreover, the interactions of these subtraits might lead to reversals in individual traits. If gazelles evolve greater agility, cheetahs might have to respond with more agility at the expense of speed, enriching the potential for more speed evolution in the future. Such trait interactions may lead to complex paths through phenotype space as in evolutionary chase arms races (Brockhurst et al., 2014). Our geometric model does not currently capture all of these processes and other kinds of models might be required to address them explicitly, but it does at least point to their importance. Mathematically joining or reconciling joint‐phenotype and separate‐phenotype models is an interesting topic for the future.

There are also other questions that could be explored by combining Fisher's geometric model with the joint phenotype concept. An obvious extension is to include correlated nonconflict traits to understand how conflict influences pleiotropic effects on other traits and the cost of complexity (Orr, 2000). We might expect evolution due to conflict to keep nonconflict traits from their optima because of pleiotropy, and as a result increase the rate of evolution of nonconflict traits.

The results here are therefore just a first step toward using the geometric model to understand conflict and arms races. But they show that Fisher's geometric model is capable of incorporating conflict and describing some of the major features long thought to be important in arms races and also generating more novel insights.

## CONFLICT OF INTEREST

We have no conflicts of interest to disclose.

## AUTHOR CONTRIBUTIONS

TS codesigned study, wrote the code, conducted the simulations, and cowrote the manuscript. DQ conceived the study, codesigned it, and cowrote the manuscript. All authors gave final approval of the manuscript.

## Data Availability

Python code for simulations is available at https://github.com/tjscott214/long-term-conflict-with-1nFGM (Scott, 2019). We include programs for simulating standard adaptation, conflict, and abiotic change along with example command line code and R code to plot results.
