# Supplementary material for: Long‐term evolutionary conflict, Sisyphean arms races, and power in Fisher's geometric model
Source: Ecol Evol. 2019 Sep 4;9(19):11243–53. doi: 10.1002/ece3.5625 (PMC6802030; doi:10.1002/ece3.5625)
Supplement: Supplementary file 1 [file ECE3-9-11243-s001.docx]

**Supplementary Figures**

Figures S1-S4 show simulation results for different mutation distributions (S1 and S2) and fitness shape parameters (S3 and S4). Figure S5 shows the effect of varying population sizes between two conflicting parties. Figures S6-S8 show simulation results when conflict parties are asymmetrical and party 1 is given higher evolutionary potential. Figures S9-S11 show asymmetry results when party 2 is given higher evolutionary potential. We note that party 2 in Figures S6-S11 has different evolutionary potential than standard adaptation and abiotic change simulations and is thus not directly comparable to them.

**Supplementary Figure Legends**

Figure S1: Uniformly distributed mutations. These results are the same as those shown in Figure 3 except with uniformly distributed mutations. Equilibrium properties of party 1’s average mutation sizes and intersection fitness (*w_0_*) under standard adaptation (yellow), conflict (blue), and abiotic change (pink) simulations with uniformly distributed mutations (from $\boldsymbol{-2}\bar{\boldsymbol{m}}$ to $\boldsymbol{2}\bar{\boldsymbol{m}}$). (A) Mean distance to the optimum. (B) Mean fitness during equilibrium. (C) Percent of mutations that are fixed during equilibrium. (D) Effect size of fixed mutations. All simulations began at the origin, have a fitness function shape parameter of 2, and a normal mutation distribution. All parameters are the same for each party for all parameter combinations shown. Means are calculated based on data collected from iteration 500 to 5,500 from 1,000 replicate simulations. Vertices show actual mean values from simulations.

Figure S2: Exponentially distributed mutations. These results are the same as those shown in Figure 3 except with exponentially distributed mutations. Equilibrium properties of party 1’s average mutation size and intersection fitness (*w_o_*) under standard adaptation (yellow), conflict (blue), and abiotic change (pink) simulations with exponentially distributed mutations with mean, $\boldsymbol{\beta=}\bar{\boldsymbol{m}}$. (A) Mean distance to the optimum. (B) Mean fitness during equilibrium. (C) Percent of mutations that are fixed during equilibrium. (D) Effect size of fixed mutations. All simulations began at the origin, have a fitness function shape-parameter of 2, and a normal mutation distribution. All parameters are the same for each party for all parameter combinations shown. Means are calculated based on data collected from iteration 500 to 5,500 from 1,000 replicate simulations. Vertices show actual mean values from simulations.

Figure S3: Stronger selection (larger shape parameter). These results are the same as those shown in Figure 3 except the shape parameter is 2. Equilibrium properties of the geometric model under varying average mutation sizes (normally distributed) and Intensity of conflict (measured as lag load at the origin/equilibrium 1-*w_0_* from fitness functions with a shape-parameter of 2). Colors indicate the version of the geometric model: standard adaptation (yellow), conflict (blue), and abiotic change (pink). (A) Mean distance to the optimum. (B) Mean fitness during equilibrium. (C) Percent of mutations that are fixed during equilibrium. (D) Effect size of fixed mutations. Means are calculated based on data collected from iteration 125 to 5,125 from 1,000 replicate simulations. Vertices show actual mean values from simulations.

Figure S4: Weaker selection (smaller shape parameter). These results are the same as those shown in Figure 3 except with a shape parameter of 1/8. Equilibrium properties of the geometric model under varying average mutation sizes (normally distributed) and intensities of conflict (measured as lag load at the origin/equilibrium 1-*w_0_* from fitness functions with a shape parameter of 1/8). Colors indicate the version of the geometric model: standard adaptation (yellow), conflict (blue), and abiotic change (pink). (A) Mean distance to the optimum. (B) Mean fitness during equilibrium. (C) Percent of mutations that are fixed during equilibrium. (D) Effect size of fixed mutations. Means are calculated based on data collected from iteration 2,000 to 7,000 from 1,000 replicate simulations. Vertices show actual mean values from simulations.

Figure S5: Fitness power of party 1 when genetic drift varies between parties. N_2_ = 10 for all simulations. N_1_ is N_2_ multiplied by the N advantage. To focus on genetic drift alone, mutational input was kept the same in populations of different sizes (one mutation per iteration). Conflict load in these simulations is 0.2 and *ω = 2, 1/2, or 1/8* as indicated in the legend. Average fitness was calculated over 5,000 iterations as indicated in the text. Simulations were repeated 2,000 times to reduce the amount of noise.

Figure S6: Party 1 has greater relative selection strength than party 2. These simulations are the same as those shown in Figure 3 except party 1 has stronger selection (*f* = 2). Colors indicate the version of the geometric model: standard adaptation (yellow), party 1 (blue), party 2 (green), and abiotic change (pink). Standard adaptation and abiotic change still have the same parameters as party 1 and the degree of abiotic change is taken from party 2 in the conflict simulation (A) Mean distance to the optimum. (B) Mean fitness during equilibrium. (C) Percent of mutations that are fixed during equilibrium. (D) Effect size of fixed mutations. Means are calculated based on data collected from iteration 500 to 5,500 from 1,000 replicate simulations. Vertices show actual mean values from simulations.

Figure S7: Party 1 has greater relative mutation size than party 2. These simulations are the same as those shown in Figure 3 except party 1 has larger mutations (κ = 2). Colors indicate the version of the geometric model: standard adaptation (yellow), party 1 (blue), party 2 (green), and abiotic change (pink). Standard adaptation and abiotic change still have the same parameters as party 1 but the degree of abiotic change is taken from party 2 in the conflict simulation. (A) Mean distance to the optimum. (B) Mean fitness during equilibrium. (C) Percent of mutations that are fixed during equilibrium. (D) Effect size of fixed mutations. Means are calculated based on data collected from iteration 500 to 5,500 from 1,000 replicate simulations. Vertices show actual mean values from simulations.

Figure S8: Party 1 has greater relative mutational input than party 2. These simulations are the same as those shown in Figure 3 except party 1 has a larger mutational input (*r* = 2). Colors indicate the version of the geometric model: standard adaptation (yellow), party 1 (blue), party 2 (green), and abiotic change (pink). Standard adaptation and abiotic change still have the same parameters as party 1 and the degree of abiotic change is taken from party 2 in the conflict simulation. (A) Mean distance to the optimum. (B) Mean fitness during equilibrium. (C) Percent of mutations that are fixed during equilibrium. (D) Effect size of fixed mutations. Means are calculated based on data collected from iteration 500 to 5,500 from 500 replicate simulations. Vertices show actual mean values from simulations.

Figure S9: Party 1 has weaker relative selection strength than party 2. These simulations are the same as those shown in Figure 3 except party 1 has weaker selection (*f* = 0.5). Colors indicate the version of the geometric model: standard adaptation (yellow), party 1 (blue), party 2 (green), and abiotic change (pink). Standard adaptation and abiotic change still have the same parameters as party 1 and the degree of abiotic change is taken from party 2 in the conflict simulation (A) Mean distance to the optimum. (B) Mean fitness during equilibrium. (C) Percent of mutations that are fixed during equilibrium. (D) Effect size of fixed mutations. Means are calculated based on data collected from iteration 500 to 5,500 from 1,000 replicate simulations. Vertices show actual mean values from simulations.


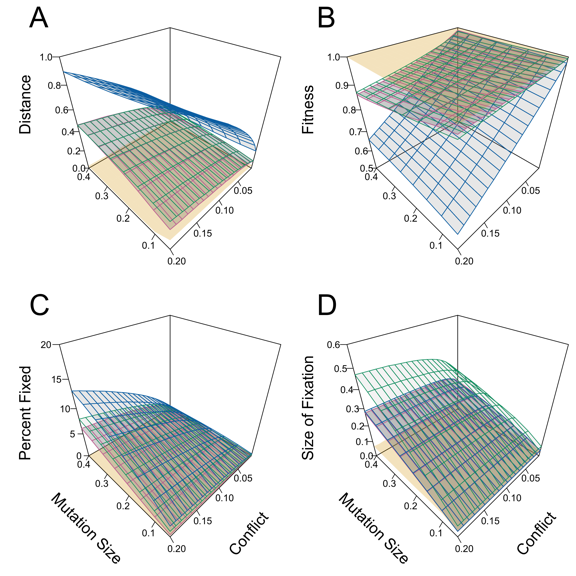


Figure S10: Party 1 has smaller relative mutation size than party 2. These simulations are the same as those shown in Figure 3 except party 1 has smaller mutations (κ = 0.5). Colors indicate the version of the geometric model: standard adaptation (yellow), party 1 (blue), party 2 (green), and abiotic change (pink). Standard adaptation and abiotic change still have the same parameters as party 1 but the degree of abiotic change is taken from party 2 in the conflict simulation. (A) Mean distance to the optimum. (B) Mean fitness during equilibrium. (C) Percent of mutations that are fixed during equilibrium. (D) Effect size of fixed mutations. Means are calculated based on data collected from iteration 500 to 5,500 from 1,000 replicate simulations. Vertices show actual mean values from simulations.


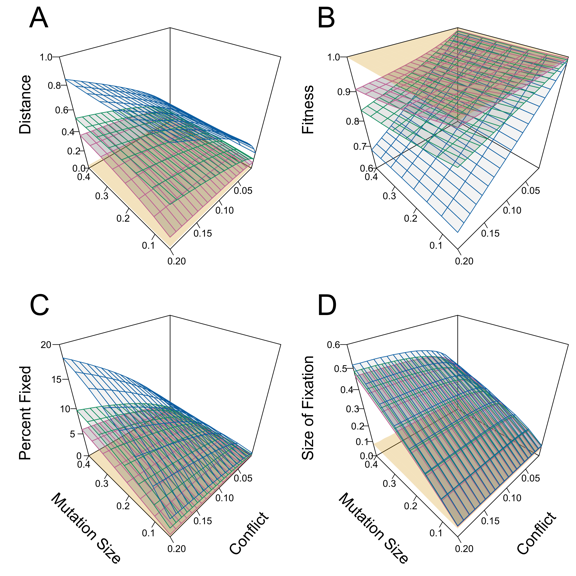


Fig. S11. Party 1 has lower relative mutational input than party 2. These simulations are the same as those shown in Figure 3 except party 1 has a lower mutational input (*r* = 0.5). Colors indicate the version of the geometric model: standard adaptation (yellow), party 1 (blue), party 2 (green), and abiotic change (pink). Standard adaptation and abiotic change still have the same parameters as party 1 and the degree of abiotic change is taken from party 2 in the conflict simulation. (A) Mean distance to the optimum. (B) Mean fitness during equilibrium. (C) Percent of mutations that are fixed during equilibrium. (D) Effect size of fixed mutations. Means are calculated based on data collected from iteration 500 to 5,500 from 500 replicate simulations. Vertices show actual mean values from simulations.
